# Blockade of the SNARE Protein Syntaxin 1 Inhibits Glioblastoma Tumor Growth

**DOI:** 10.1371/journal.pone.0119707

**Published:** 2015-03-24

**Authors:** Fausto Ulloa, Alba Gonzàlez-Juncà, Delphine Meffre, Pablo José Barrecheguren, Ramón Martínez-Mármol, Irene Pazos, Núria Olivé, Tiziana Cotrufo, Joan Seoane, Eduardo Soriano

**Affiliations:** 1 Department of Cell Biology, University of Barcelona, Parc Cientific de Barcelona, 08028, Barcelona, Spain; 2 Centro de Investigación Biomédica en Red sobre Enfermedades Neurodegenerativas (CIBERNED), ISCIII, 28031, Madrid, Spain; 3 Translational Research Program, Vall d'Hebron Institute of Oncology (VHIO), Vall d’Hebron University Hospital, 08035, Barcelona, Spain; 4 Universitat Autònoma de Barcelona, 08193, Cerdanyola del Vallès, Spain; 5 Institute for Research in Biomedicine (IRB), Cell and Developmental Biology Program, Barcelona, 08028, Spain; 6 Vall d´Hebron Institute of Research (VHIR), 08035, Barcelona, Spain; 7 Institució Catalana de Recerca i Estudis Avançats (ICREA), 08010, Barcelona, Spain; Swedish Neuroscience Institute, UNITED STATES

## Abstract

Glioblastoma (GBM) is the most prevalent adult brain tumor, with virtually no cure, and with a median overall survival of 15 months from diagnosis despite of the treatment. SNARE proteins mediate membrane fusion events in cells and are essential for many cellular processes including exocytosis and neurotransmission, intracellular trafficking and cell migration. Here we show that the blockade of the SNARE protein Syntaxin 1 (Stx1) function impairs GBM cell proliferation. We show that Stx1 loss-of-function in GBM cells, through ShRNA lentiviral transduction, a Stx1 dominant negative and botulinum toxins, dramatically reduces the growth of GBM after grafting U373 cells into the brain of immune compromised mice. Interestingly, Stx1 role on GBM progression may not be restricted just to cell proliferation since the blockade of Stx1 also reduces *in vitro* GBM cell invasiveness suggesting a role in several processes relevant for tumor progression. Altogether, our findings indicate that the blockade of SNARE proteins may represent a novel therapeutic tool against GBM.

## Introduction

Glioblastoma is the most common type of primary brain tumor in adults. Despite significant advances in the understanding of the molecular and cellular basis of tumor origin and progression, GBM is usually fatal, with a median overall survival of 15 months from diagnosis despite of the treatment [1,2]. SNARE proteins are essential for many cellular events requiring membrane fusion, including exocytosis and neurotransmission, intracellular trafficking, and cell proliferation [3,4]. They have been classically divided in two groups: t-SNAREs (including syntaxin and SNAP family proteins) and v-SNAREs (including Vamp family proteins), depending on whether they are located at the target membrane or the donor vesicle respectively. In the nervous system, the t-SNAREs SNAP25 and Stx1 and the v-SNARE VAMP2 are required for calcium-dependent exocytosis and neurotransmitter release [5]. Recent findings have implicated several SNARE proteins, particularly Stx1, in axonal growth and neuronal migration [6,7,8]. Two main Stx1 isoforms have been recognized (Stx1a and Stx1b) which display a differentiated pattern of expression [9]. Interestingly, Stx1 is expressed in several tumors, including small cell lung carcinoma and the most aggressive forms of colorectal cancer [10,11]. Given that SNARE proteins are involved in neuronal migration and GBM is a very invasive tumor, here we examined whether the blockade of the exocytotic machinery, in particular Stx1 inactivation, has an impact on the growth and progression of GBM *in vivo*.

## Material and Methods

### Cells

GBM U251, C52, C4, hs683, T98G, A172, U87 and U373 cells were obtained from ATCC. All the cells were cultured in DMEM supplemented with 10% FBS at 37°C and 5% CO2. GFP and the rat Stx1a H3TM domain fused with EGFP cloned into a pEGP vector [7] were transiently transfected by using the Neon Transfection System (Invitrogen) according to the manufacturer’s instructions. To establish U373 populations stably expressing a GFP-firefly luciferase fusion protein, cells distributed in six 10 cm dishes were transfected with pEGFP-Luc vector (Promega) using the Lipofectamine Plus system (Invitrogen) and following the manufacture’s instructions. Cells were then selected in normal media supplemented with 500 ug/ml G418 and positive GFP cells were isolated from the surviving cells by two rounds of FACS. U373 GFP-luciferase-expressing cells were then infected with lentiviral particles codifying for scrambled or specific shRNA human Stx1a sequences (SMARTchoice Lentiviral Human shRNA particles were purchased from Dharmacon: Stx1a-Sh01: TCCGACATGACCTCCACAA; Stx1a-Sh02: TACTCGATCCTGTCAATCA). An independent population of U373 GFP-luciferase cells was infected with retroviral particles codifying for the H3TM domain of Stx1a. To this end, the sequence of the rat Stx1a H3TM domain fused with EGFP was subcloned into pBabe puro vector (a gift from R. Gomis, IRB Barcelona) and retroviral particles were prepared. The resulting cell populations showed similar levels of luciferase activity compared to their corresponding controls.

### Western blot

Subconfluent cultured cells were lysed with lysis buffer (10mM Tris, pH 7, 500mM NaCl, 1mM EDTA, 1mM EGTA, 1% Triton X-100, 0.5% NP-40) supplemented with Complete Protease Inhibitor Cocktail (Roche) and the protein extract was fractioned by SDS-PAGE. Stx1 WB was performed as previously described [7]. Primary antibodies used for the immunoblot detection were mAb Munc18a (BD, Biosciences), mAb HPC1 (Sigma) for Stx1, rabbit anti-Stx2 (Synaptic Systems), mAb anti-Stx3, clone 1–146 (Merck Millipore), rabbit anti-Stx4 (Synatpic Systems), rabbit anti-Vamp2 (Synaptic Systems), mAb SMI-81 (Sternberger Meyer) for SNAP25, mAb 1501 (Merck Millipore) for actin, and mAb D66 (Sigma) for tubulin. Appropriate secondary antibodies were obtained from DAKO.

### *In vitro* cell invasion assay

20 x 10^5^ cells were cultured in DB BioCoat Matrigel Invasion Chambers (BD Biosciences) in DMEM 0.5% FBS. In the lower chamber DMEM 10% FBS was added. After 24 h of incubation cells were fixed with 4% paraformaldehyde (PFA) in PBS and non-invasive cells were removed with a cotton swab. Cell nuclei were stained with DAPI and images were collected with a fluorescence microscope. Cell counting was done by using ImageJ software (NIH). Cell invasion assays were performed in triplicate a minimum of three times.

### Brain tumor xenografts

All mouse experiments were approved and performed in accordance with the guidelines of the Institutional Animal Care Committee of the Vall d'Hebron Research Institute in agreement with the European Union and national directives. 1 x 10^6^ cells were stereotactically inoculated into the corpus striatum of the right brain hemisphere (1 mm anterior and 1.8 mm lateral to the bregma; 2.5 mm intraparenchymal) of 9-week-old athymic Nude-*Foxn1* ^*nu*^ mice (Charles River Laboratories). Cells inoculated in the presence of BoNT/C1 (generously provided by M.R. Popoff, Institut Pasteur, Paris, France) were pretreated with the toxin (375 pg of toxin) 10 minutes before inoculation and then co-injected. Eight mice per experimental condition were used. Mice were euthanized when they presented neurological symptoms or a significant weight loss. In order to estimate the size of tumors the luciferase activity of inoculated tumor cells was quantified in a Xenogen-CCD camera from IVIS. Magnetic resonance imaging (MRI) analysis was performed and images were acquired using 9.4 T vertical bore magnet interfaced to an AVANCE 400 system (Bruker). Under anesthesia by xylazine/ketamine, mice were given an intraperitoneal injection of gadolinium diethylenetriamine penta-acetic acid at a dose of 0.25 mmol gadolinium/kg body weight and placed in the radio frequency coil (inner diameter 35 mm). After localizer imaging on three orthogonal axes, T1-weighted images of the entire mouse brain were acquired using a spin echo sequence with TR and TE set to 800 and 5.7 ms, respectively. Tumor size was quantified by measuring the number of pixels corresponding to tumor tissue in each image using the software provided by the manufacturer (Bruker). When the tumor was visible in more than one image, areas corresponding to tumor tissue were measured together.

### BrdU incorporation analysis

For *in vitro* analysis unsynchronized cultures with an equivalent number of cells were incubated in media supplemented with 0.05 mM of BrdU during 90 min at 37°C. Then, cells were fixed with PFA 4% 10 min at room temperature and processed for the immunodetection of BrdU (see below). All *in vitro* BrdU incorporation analysis were done in triplicate a minimun of three times. For *in vivo* analysis, a single dose of BrdU (100 mg/Kg in PBS) was intraperitoneally injected to the animals 1 hour prior to their perfusion. Mice were perfused with 4% PFA in PBS. The brains were dissected out, post-fixed in 4% PFA, cryoprotected in 30% sucrose, and frozen in cold methyl-butanol. BrdU and GFP double-immunostaining was performed in 40 μm serial cryosections of at least three animals per condition. For immunostaining, the BrdU antigen was exposed by incubating the sections in 1N HCl at 45°C for 30 min. A rat anti-BrdU (AbD Serotec) and a rabbit anti-GFP (Invitrogen) were used as primary antibodies. Sections were immunoreacted for 48h at 4°C, and then incubated with secondary antibodies for 2 hours at room temperature. After washing, sections were mounted with Mowiol. Confocal images were acquired and the counting of BrdU- and GFP-positive cells was performed using ImageJ software (NIH).

### Cell cycle flow cytometry analysis and double nucleated cell quantification

Non synchronized cell cultures were individualized by trypsinization and fixed with cold 70% EtOH. Cell DNA was stained with a 40 ug/ml propidium iodide—100 ug/ml RNAse solution and cell cycle profiles were obtained by the Scientific technique services of the University of Barcelona. For the determination of the proportion of binucleated cells, an equivalent number of cells were seeded on coverslips and after 24 h they were stained with and anti-tubulin antibody and Dapi (Sigma). Conventional epifluorescence images were obtained acquired and the counting of binucleated cells was performed using ImageJ software (NIH).

### Statistics

Statistical analyses were performed using the GraphPad Prism 5.03 software. Student’s T-test was used to compare means in the in vitro assays. Mann-Whitney test was used for experiments with animals. Data in graphs are expressed as mean +/- SEM. In graphics one star means a *p* value < 0.05, two stars means a *p* value < 0.01 and three stars a *p* value < 0.001.

## Results and Discussion

### Impairment of Stx1 function reduces proliferation and invasion capacity of GBM cells *in vitro*

**Fig 1:**
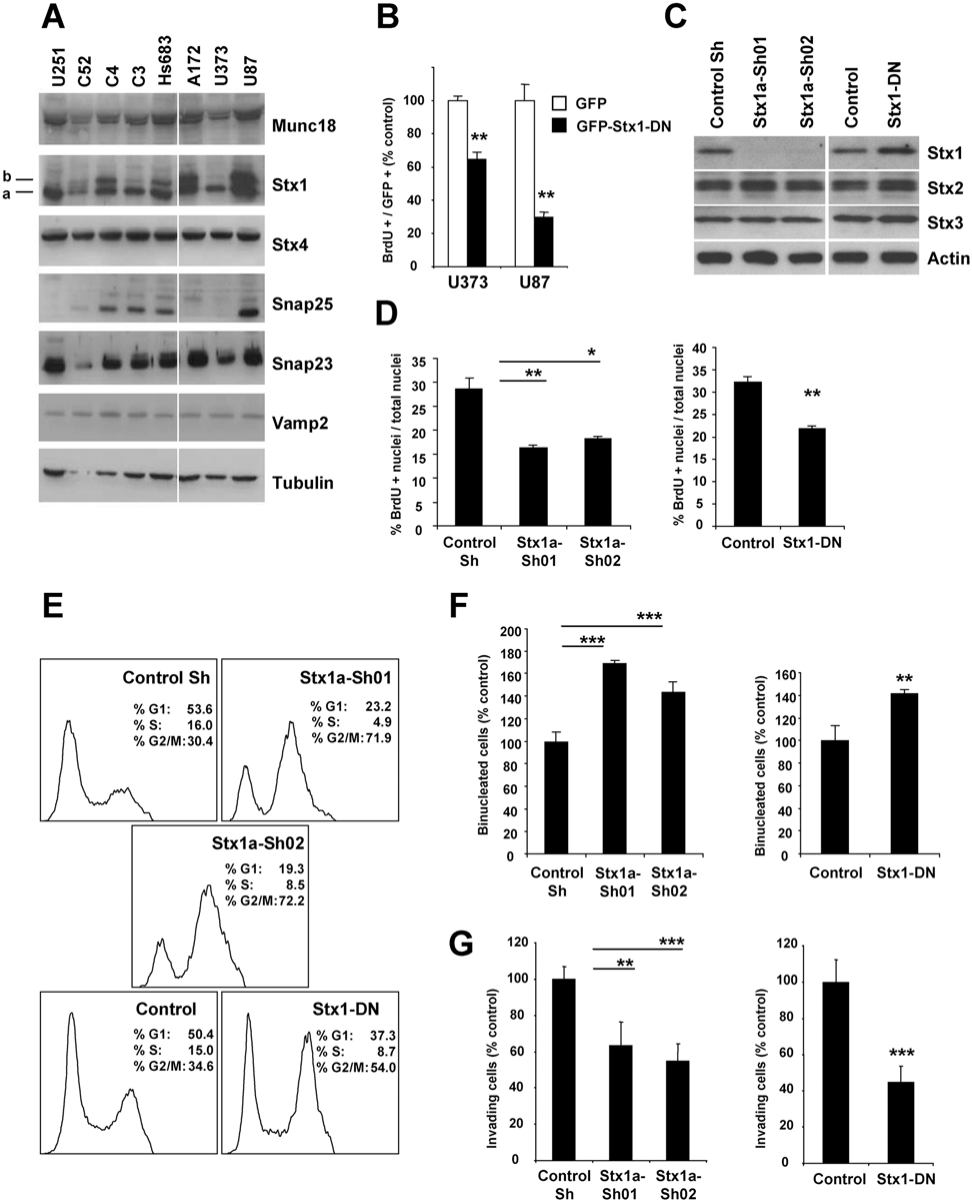
Blockade of Stx1 function reduces GBM cell proliferation *in vitro*. **A.** Expression of exocytic SNARE machinery in several established GBM cell lines determined by immunoblot. **B.** Quantification of BrdU-positive nuclei in GBM cells. The transient expression of a GFP fused Stx1 dominant negative form (Stx1-DN) reduces the rate of BrdU incorporation in GBM cells (** p ≤ 0.01). Figure shows representative results of three independent experiments. **C.** U373 GBM cells with a reduced expression of Stx1 were established by ShRNA lentiviral transduction strategies. Immunoblot showing Stx1 downregulation in U373 cell populations stably expressing two Stx1a ShRNA (Sh01 and Sh02) sequences. Stx2 and Stx3 expression levels are not modified in U373 cells with their Stx1 function blocked (U373 Stx1a-Sh01/Sh02 and Stx1-DN). **D.** Quantification of BrdU incorporation in U373 GBM cells. Cells with their Stx1 function stably blocked showed a minor rate of BrdU incorporation (* p ≤ 0.05; ** p ≤ 0.01). Figure shows representative results of three independent experiments. **E.** Cell cycle profile of unsynchronized U373 GBM cells obtained by flow cytometry. The stable blockade of Stx1 function results in an increment of cell populations at G2/M. **F.** Quantification of the frequency of binucleated U373 GBM cells. Cells with their Stx1 function stably blocked show a major proportion of binucleated cells than their respective controls. **G.** Boyden chamber invasion assay of the indicated U373 cells, showing that loss-of-function of Stx1 reduces U373 cell invasion capacity (** p ≤ 0.01; *** p ≤ 0.001). Figure shows representative results of three independent experiments.

We first determined the expression of SNARE proteins in various GBM cell lines. Consistent with previous reports [12], the expression of several SNAREs was detected in GBM cell populations, including Stx1, Stx4, Vamp2, SNAP25, SNAP23, and of the regulatory protein Munc18 (Fig. 1A). As shown in Fig. 1A, GBM cell populations express different Stx1 isoforms. The function of all the Stx1 isoforms can be blocked by the action of a dominant negative form consisting of the H3TM (H3: SNARE, TM: transmembrane) domains of Stx1a (Stx1-DN) [7]. The overexpression of the Stx1-DN form in two GBM cells (U373 and U87) that show a distinct pattern of Stx1 isoforms expression reduced statistically significant the rate of BrdU incorporation. (Fig. 1B). Anti- active Caspase3 staining did not reveal any induction of apoptosis (data not shown). We selected U373 cells for further experimental manipulation. After inoculation in brains of immunocompromised mice these cells develop invasive tumors that resemble those observed in patients. We generated stable U373 cell lines (expressing a GFP-Luciferase fusion protein, GFP-Luc) with down-regulation of Stx1a by using lentiviral vectors (Stx1a-Sh) (Fig. 1C). We also established a U373 cell line expressing the Stx1a dominant negative form (U373 Stx1-DN) [7,13]. The blockade of Stx1 did not produce major modifications on the expression levels of other related syntaxins as Stx2 and Stx3 (Fig. 1C). Then, we determined the impact of Stx1 inactivation on the properties of U373 cells *in vitro*. As previously observed, the stable blockade of Stx1 function reduced approximately between 32 to 43% the rate of BrdU incorporation in U373 cells (Fig. 1D). Flow cytometry analysis of cell cycle revealed that the cell populations with their Stx1 function blocked show a higher proportion of cells (approx. 19–42%) in G2/M than their corresponding control cells (Fig. 1E). This observation is compatible with previously published data indicating that the action of several SNARE proteins, particularly Stx1 in some invertebrates and Stx2 and Stx16 in mammalian cells, is important in cytokinesis [14,15]. Consistent with this scenario, we observed a higher proportion (40–70%) of binucleated cells when Stx1 function was blocked (Fig. 1F). In addition, the blockade of Stx1 markedly reduced the migration capacity of U373 cells as tested in Boyden chambers, compared to control cells (Fig. 1G). These findings are consistent with previous data showing that the down-regulation of Vamp3, Stx13 and Snap23 reduces cell invasion of HT-1080 fibrosarcoma cells *in vitro*, and they support a relevant role of SNARE proteins in tumor cell migration [16]. Altogether, these results indicate that the disruption of Stx1 function impairs several processes in glioblastoma cells.

### Impairment of the Stx1 function results in the reduction of GBM tumor size *in vivo*

**Fig 2:**
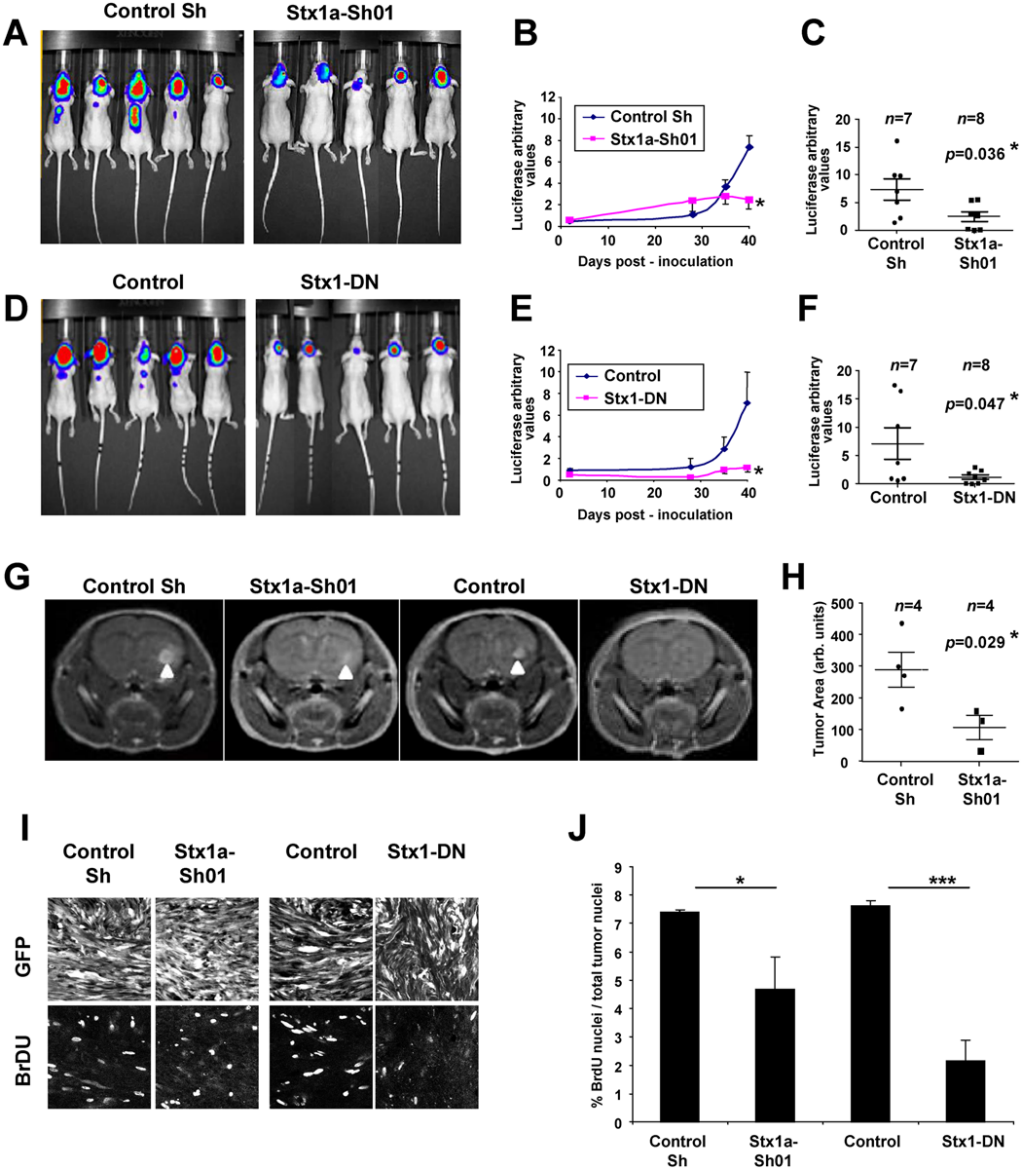
Disruption of the Stx1 function impairs GBM tumor progression *in vivo*. An equal number of the indicated U373 cell populations were stereotactically inoculated into the brain of athymic nude mice. The size of the tumors was estimated at different days post-inoculation by the quantification of luciferase activity in the tumor cells. **A, D.** Representative images of the luciferase signal from mice inoculated with the indicated U373 GBM cells after 40 DPI. **B, E.** Growth curves of the indicated U373 GBM tumors showing a marked reduction of the tumor sizes after impairment of Stx1 function (* p ≤ 0.05 at 40 DPI). **C, F.** Scatter plots showing the individual size of the indicated U373 GBM tumors after 40 DPI. **G.** Representative entire brain NMR images of mice inoculated with the indicated U373 cell populations after 30 DPI (arrowheads indicate the tumors formed) showing a marked reduction in the Stx1a-Sh01 and Stx1-DN cells. **H.** Scatter plot showing the area of the indicated U373 GBM tumors formed after 30 DPI. **I.** Representative confocal images from histological sections of 30 DPI brain tumors stained with anti-GFP and anti-BrdU antibodies. **J.** Quantification of BrdU-positive nuclei in 30 DPI U373 cell tumors showing that Stx1 loss-of-function reduces proliferation (* p ≤ 0.05; *** p ≤ 0.001).

To study whether Stx1 loss-of-function affects the progression of GBM *in vivo*, we performed intracerebral xenotransplantation experiments using U373 GFP-Luc cell lines [17] and quantified the rate of tumoral growth over time by detecting luciferase activity. Tumors derived from control U373 cells exhibited a moderate growth until 30 days post-inoculation (30DPI) followed by an exponential growth that reached a maximum at 40DPI (Fig. 2A-F). In contrast, tumors derived from U373 Stx1a-Sh01 and U373 Stx1-DN cells showed a dramatic reduction in size at 40DPI, being 3–7 times smaller than control tumors (Fig. 2A-F).

To confirm these findings, we monitored U373 cell xenografts by means of magnetic resonance imaging (MRI). We confirmed that U373 Stx1a-Sh01 cells formed smaller tumors than controls (Fig. 2G,H), while U373 Stx1-DN tumors were practically undetectable (data not shown). Altogether, these results show that distinct Stx1 loss-of-function approaches consistently lead to a dramatic reduction in the progression of experimental GBM *in vivo*. To determine whether Stx1 loss-of-function alters proliferation *in vivo* as previously seen *in vitro*, nude mice, grafted with U373 Stx1a-Sh01 and U373 Stx1-DN cells for 30 days, were injected with BrdU one hour before euthanasia and their brains processed for BrdU-immunohistochemistry. Quantification of GFP/BrdU-labeled U373 cells revealed a ∼40–70% reduction in the percentage of BrdU+ U373 Stx1a-Sh01 and Stx1-DN cells compared to U373 control cells (Fig. 2I,J).

**Fig 3:**
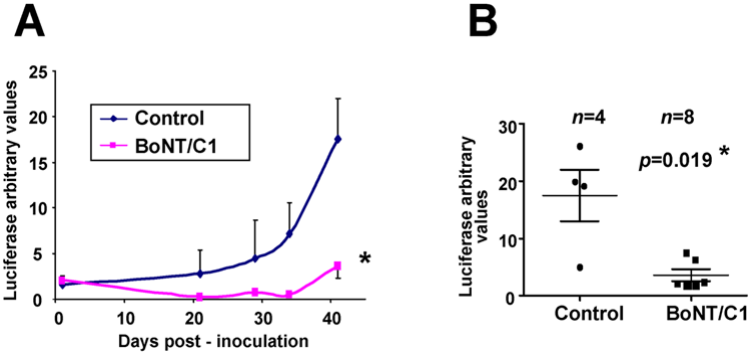
Botulinum toxin C1 impairs glioblastoma progression in vivo. **A, B.** U373 cells were inoculated into the brain of nude mice in the absence or in the presence of botulinum toxin C1 (BoNT/C1), which specifically cleaves the SNARE proteins Stx1a/b and SNAP25. **(A)** Growth curve of GBM tumors determined by luciferase quantification (* p ≤ 0.05 at 40 DPI), and **(B)** scatter plot showing the individual size of the U373 GBM tumors formed after 40 DPI, demonstrating a marked reduction of GBM after BoNT/C1 treatment.

Our results suggest a scenario where it may be possible to impair the growth of glioblastoma tumors by using drugs which block Stx1 activity. Botulinum neurotoxins (BoNTs) are potent and specific inhibitors of SNARE functions. This action is achieved by cleaving and inactivating selective SNARE proteins [18]. Given their specificity and long-life action, BoNTs are widely used in medical practice [19]. To examine whether BoNTs can also reduce glioblastoma tumoral progression, we co-injected U373 cells with BoNT/C1, which cleaves Stx1 [20]. After 40DPI, U373 cell tumors were about five times smaller in mice inoculated with BoNT/C1 than in untreated, control mice (Fig. 3A,B). In addition to Stx1, Stx2 and Stx3 are targets of BoNT/C1 [20]. Since all of these syntaxins are expressed in U373 cells (Fig. 1C), it remains to be elucidated whether the effect of BoNT/C1 on U373 GBM growth is due exclusively to its action on Stx1. Nevertheless, this result supports the feasibility of using Stx1 blocking drugs as therapeutic agents to treat GBMs.

In summary, we show here that the blockade of the SNARE protein Stx1 through three different approaches, including knock-down of Stx1a, expression of the H3TM dominant negative form of Stx1a and inhibition of Stx1 by treatment with BoNT/C1, consistently results in a dramatic decrease in glioblastoma tumor progression in an orthotopic mouse model. This reduction ranged from 3 times (in Stx1a-Sh01 cells) to 7 times in the experiments with U373 cells expressing the H3TM construct. To our knowledge, this study is the first to describe that the inactivation of SNARE proteins has an important impact on the growth of glioblastoma tumors. Our data show that Stx1 loss-of-function results in a decrease in the proliferation of GBM tumoral cells. However, as SNARE proteins, and particularly Stx1, are involved in many other key functions, including migration, exocytosis, protein secretion and adhesion, in addition to cell proliferation, it is likely that targeting a single SNARE protein may simultaneously dysregulate several of the above processes, all of them crucial for tumor progression [21,22,23]. Consistent with this idea, we have shown here that the blockage of Stx1 also affects GBM cells invasion *in vitro* (Fig. 1G).

Our findings in experimental GBM *in vivo* model open up the possibility of using Stx1 or other SNAREs as potential therapeutic targets. However, in order to be of clinical interest, our data should be replicated in other GBM models. It is interesting to note that BoNTs, the most potent and selective inhibitors of SNARE proteins, are extensively used in medical practice, particularly to treat acute pain, muscular spasms, and Parkinson’s disease [19]. Moreover, recent studies report the shrinkage of prostatic and gastric tumors after the injection of BoNT/A [24,25]. The main drawback associated with a treatment based on the use of inhibitors of SNARE proteins is toxicity, since neurotransmission can be seriously affected. However, this problem could be overcome by employing specific peptides which just inhibit the formation of the SNARE complexes vital for the progression of glioblastomas, but not those involved in other cellular functions. It is known that differences in the combination/configuration of SNARE protein complexes explain the diversity of exocytic processes [26]. Thus, it may be possible to block Stx1 actions on GBM progression without the secondary toxic effects caused by an inhibition of neurosecretion. Even though future research is clearly required to identify Stx1 loss of function mechanism of action, our data supports the hypothesis that targeting SNARE proteins may be an effective tool to reduce GBM tumor growth *in vivo*, by simultaneously targeting several cellular processes relevant to cancer growth and progression.
